# Supplementary material for: Whole genome resequencing reveals diagnostic markers for investigating global migration and hybridization between minke whale species
Source: BMC Genomics. 2017 Jan 13;18:76. doi: 10.1186/s12864-016-3416-5 (PMC5237217; doi:10.1186/s12864-016-3416-5)
Supplement: Additional file 1:​ — Probabilities for heterozygote genetic markers in hybrids﻿. (PDF 29 kb) [file 12864_2016_3416_MOESM1_ESM.pdf]

# Probabilities for heterozygote genetic markers in hybrids

Ketil Malde and Hans J. Skaug

March 18, 2016

Given two populations and genetic markers that separate them, we show how to calculate the frequencies of specific genotypes in hybrids. In particular, we are interested in the case where a migrant enters a native population and interbreeds with it, and where the offspring continues to interbreed with the native population, resulting in new generations of back-crossed offspring.

## 1 Assuming fully diagnostic markers

A marker is fully diagnostic if no allele occurs in both populations, and, restricting analysis to two alleles, all non-hybrid individuals are homozygote. We label the native allele $A$, and the foreign allele $a$. We then get probabilities for the different genotypes given in Table 1 below.

Table 1: Genotype probabilities with fully diagnostic markers for increasing generations of back-crossed hybrids.

| BC Gen | P(AA) | P(Aa) | P(aa) |
|---|---|---|---|
| migrant | 0 | 0 | 1 |
| native | 1 | 0 | 0 |
| F1 | 0 | 1 | 0 |
| F2 | 0.5 | 0.5 | 0 |
| F3 | 0.75 | 0.25 | 0 |
| : | : | : | : |
| Fn | $1\text{-}2^{1\text{-}n}$ | $2^{1\text{-}n}$ | 0 |

An F1 hybrid by necessity inherits one allele from each population, and thus it is always heterozygote. An F2 back cross inherits one allele from the native population, and one from the F1 hybrid. The probability of heterozygosity is therefore the probability of inheriting the foreign allele ($a$) from the hybrid, i.e. 0.5. Similarly, the probability of homozygosity is the probability of inheriting the native allele ($A$), also 0.5. In general, the probability of retaining the foreign allele is halved for each subsequent back-cross, leading to the formula given for the $n$th generation back-cross.

## 2 Arbitrary allele frequencies

Although markers may be fully diagnostic, n practice the foreign allele may occur in the native population, and vice versa. In any case, with limited testing we cannot ascertain that the markers are fully diagnostic; at best, we can give a confidence interval for the minor allele frequency.

To address this, rather than the actual allele values, let instead $A$ represent an allele with native origin, and let $a$ represent an allele with foreign origin. We see that we can then use Table 1 to determine the probability of a back-cross having two alleles from the native population, or retaining one allele from its migrant forebear.

Table 2: Definition of allele frequencies in the two population.

| | B | b |
|---|---|---|
| native | $p_n$ | $q_n$ |
| foreign | $q_f$ | $p_f$ |

We assume alleles B and b, and define allele frequencies as in Table 2.

From Table 3, the probability for a heterozygote in an Fn hybrid is therefore:

$$Pr(Bb) = (1 - 2^{1-n})2p_nq_n + 2^{1-n}(p_np_f + q_nq_f)$$

Table 3: The probability of the two cases of allele heritage, and the associated probabilities for the possible genotypes.

| Case | **Probability** | Genotype BB | **Genotype Bb** | Genotype bb |
|---|---|---|---|---|
| Two native alleles | $1 - 2^{1-n}$ | ${p_n}^2$ | $2p_n q_n$ | ${q_n}^2$ |
| One native, one foreign | $2^{1-n}$ | $p_n q_f$ | $p_n p_f + q_n q_f$ | $q_n p_f$ |

If we assume that the population has the same minor allele frequency, that is $p_n = p_f = p$ and $q_n = q_f = q = 1 - p$. From the relationship $p = 1 - q$, it follows that $p^2 + q^2 = p^2 + (1-p)^2 = 2p^2 - 2p + 1 = 1 - 2pq$, and we get:

$$
\begin{aligned}
Pr(Bb) &= (1 - 2^{1-n})2pq + 2^{1-n}(p^2 + q^2) \\
&= (1 - 2^{1-n})2pq + 2^{1-n}(1 - 2pq) \\
&= 2pq - 2^{1-n}2pq + 2^{1-n} - 2^{1-n}2pq \\
&= 2pq(1 - 2^{2-n}) + 2^{1-n}
\end{aligned}
$$

We observe here that if $p = 1$, the probability of a heterozygote is $2^{1-n}$, as in Table 1, and as $n$ increases, the heterozygote probability converges to $2pq$, the heterozygote probability in the native population. Table 3 gives the heterozygote probabilities for back-cross generations F1 to F10 under various minor allele frequencies.

Table 4: Table of heterozygote probabilities given generation and MAF

| Gen | 0.1 | 0.05 | 0.025 | 0.01 | 0 |
|---|---|---|---|---|---|
| 1 | 0.820 | 0.905 | 0.951 | 0.980 | 1.000 |
| 2 | 0.500 | 0.500 | 0.500 | 0.500 | 0.500 |
| 3 | 0.340 | 0.298 | 0.274 | 0.260 | 0.250 |
| 4 | 0.260 | 0.196 | 0.162 | 0.140 | 0.125 |
| 5 | 0.220 | 0.146 | 0.105 | 0.080 | 0.063 |
| 6 | 0.200 | 0.120 | 0.077 | 0.050 | 0.031 |
| 7 | 0.190 | 0.108 | 0.063 | 0.035 | 0.016 |
| 8 | 0.185 | 0.101 | 0.056 | 0.027 | 0.008 |
| 9 | 0.183 | 0.098 | 0.052 | 0.024 | 0.004 |
| 10 | 0.181 | 0.097 | 0.051 | 0.022 | 0.002 |
| native | 0.180 | 0.095 | 0.049 | 0.020 | 0.000 |

## 3 Fixed markers in the native population

For the minke whale, we find some cases of non-zero minor allele frequency in the Antarctic population, but not in the common minke population. One possible explanation can be that the larger Antarctic population allows the maintenance of a wider genetic diversity. Since we are primarily interested in the introgression of (foreign) Antarctic minke into (native) common minke populations, we might assume that $p_n = 1$ and $q_n = 0$. In that case, we can simplify (1) and the probability of heterozygotes becomes:

$$Pr(Bb) = 2^{1-n} p_f$$

## 4 Approximation with low minor allele frequency

When $q$ is small, we can make the following approximation:

$$
\begin{aligned}
Pr(Bb) &= 2pq(1 - 2^{2-n}) + 2^{1-n} \\
&= 2(1-q)q(1 - 2^{2-n}) + 2^{1-n} \\
&\approx 2q(1 - 2^{2-n}) + 2^{1-n}
\end{aligned}
$$
